# Cross-reactive antibodies binding to H4 hemagglutinin protect against a lethal H4N6 influenza virus challenge in the mouse model

**DOI:** 10.1080/22221751.2018.1564369

**Published:** 2019-01-21

**Authors:** Fatima Amanat, Philip Meade, Shirin Strohmeier, Florian Krammer

**Affiliations:** a Department of Microbiology, Icahn School of Medicine at Mount Sinai, New York, NY, USA; b Graduate School of Biological Sciences, Icahn School of Medicine at Mount Sinai, New York, NY, USA

**Keywords:** Influenza virus, hemagglutinin, H4N6, avian influenza, monoclonal antibodies

## Abstract

Influenza viruses of the H4 subtype are widespread in wild birds, circulate in domestic poultry, readily infect mammals, and tolerate the insertion of a polybasic cleavage site. In addition, serological evidence suggests that humans working with poultry are exposed to these viruses. While H4 viruses are not of immediate pandemic concern, there is a lack of knowledge regarding their antigenicity. In order to study viruses of the H4 subtype, we generated and characterized a panel of antibodies that bind a wide variety of H4 hemagglutinins from avian and swine isolates of both the Eurasian and North American lineage. We further characterized these antibodies using novel recombinant H4N6 viruses that were found to be lethal in DBA/2J mice. Non-neutralizing antibodies, which had activity in an antibody dependent cell-mediated cytotoxicity reporter assay *in vitro,* protected mice against challenge *in vivo*, highlighting the importance of effector functions. Our data suggest a high degree of antigenic conservation of the H4 hemagglutinin.

## Introduction

Influenza A viruses are widespread and are commonly found in nature since their natural reservoirs are wild birds and waterfowl. They are classified based on their two surface glycoproteins; hemagglutinin (HA) and neuramindase (NA). Sixteen subtypes of HA, ranging from H1 to H16, are found in the avian reservoir while two additional subtypes, H17 and H18, have been isolated from bats [1]. Subtypes such as H1N1, H2N2, and H3N2 have caused pandemics in humans in the past and H1N1 and H3N2 are still circulating in the human population as seasonal influenza viruses [2]. Other subtypes such as H5N1, H5N6, H6N1, H7N2, H7N3, H7N7, H7N9, H9N2, H10N7 and H10N8 have also caused zoonotic infections and might have some pandemic potential [3]. The ability to efficiently replicate in mammalian hosts is a pre-requisite for influenza viruses to cause zoonotic infections or pandemics. Several factors contribute to this ability, including the binding affinity of the viral HA to different sialic acid receptors [4]. Many avian influenza virus HAs preferentially bind to α-2,3-linked sialic acids, which are prevalent in the avian gastrointestinal tract, while human/mammalian-adapted influenza virus HAs (with exceptions) favourably interact with α-2,6-linked sialic acid receptors which are found in the human upper respiratory tract [5]. In addition, while avian influenza viruses are typically low pathogenic viruses, an introduction of a polybasic sequence in the protease cleavage site between the HA1 and HA2 subunits can lead to a highly pathogenic phenotype in birds [6] resulting in viruses that can spread systemically in infected animals. This is of concern since highly pathogenic avian influenza (HPAI) viruses like H5N1, H5N6 and H7N9 can also have high case fatality rates in humans [7–9].

Influenza viruses of the H4 subtype are considered to be low pathogenic avian influenza (LPAI) viruses and have been circulating worldwide in avian species [10,11]. They have also been detected in domestic poultry [12,13] and are capable of replicating in pigs, seals, musk beavers and other mammals [14–18]. An extensive characterization of various H4 isolates demonstrated that H4 viruses can replicate in mice without any prior adaptation [12,19] and the virus also transmitted amongst guinea pigs via direct contact [13]. In addition, most isolates bind both α-2,3-linked sialic acid receptors as well as α-2,6-linked sialic acid receptors which are found in mammals [13]. This ability to bind both types of sialic acid receptors might explain the extensive host range of H4 viruses [13,20]. A preference for α-2,6-linked sialic acid receptors is common in isolates from pigs [20,21]. Serological data shows that humans coming in close contact with poultry (e.g. farm workers) can seroconvert to H4 viruses, potentially through subclinical infection [22,23]. Furthermore, the H4 HA has been shown to be one of the subtypes to tolerate the insertion of a polybasic cleavage site which, leads to an HPAI phenotype [24–26]. It is therefore surprising that very few studies regarding the antigenic characteristics and antigenic conservation of the H4 subtype have been conducted. Here, we describe the binding breadth and *in vitro* and *in vivo* functionality of a panel of eight monoclonal antibodies (mAbs) that were generated against the head domain of the H4 HA of the prototypic isolate, A/duck/Czechoslovakia/1956 (H4N6).

## Results

### Anti-H4 antibodies bind broadly to avian and mammalian H4 HAs from both the Eurasian and North American lineage

**Figure 1.**
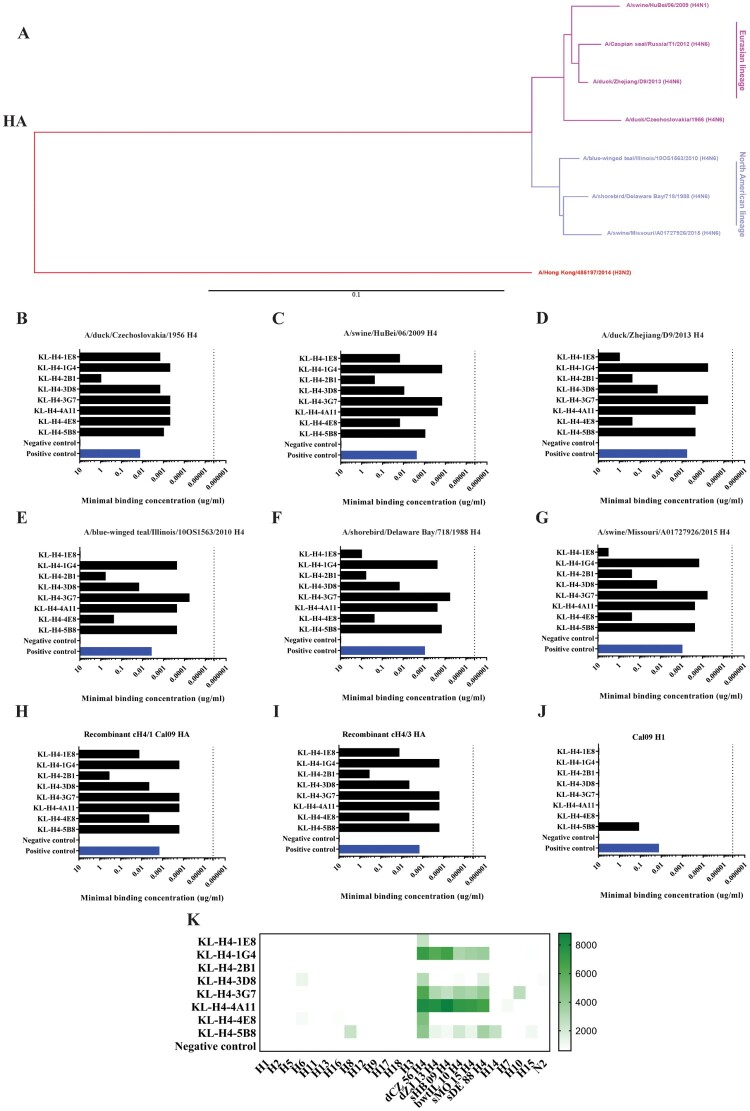
Cross-reactive antibodies bind divergent HAs from avian and mammalian isolates of both the Eurasian and North American H4 lineage. (A) Phylogenetic analysis of various selected H4 HAs. The HAs of H4 viruses cluster into two groups, Eurasian or North American lineage. The scale bar represents a 1% difference in amino acid sequence. (B–G) Binding of mAbs to recombinant H4 proteins of avian or mammalian origin. Standard ELISA assays were performed to test the binding of the eight mAbs to (B) A/duck/Czechoslovakia/1956 H4, (C) A/swine/HuBei/06/2009 H4, (D) A/duck/Zhejiang/D9/2013 H4, (E) A/blue-winged teal/Illinois/10OS1563/2010 H4, (F) A/shorebird/Delaware Bay/718/1988 H4, and (G) A/swine/Missouri/A01727926/2015 H4. (H–I) Binding of mAbs to recombinant chimeric HA proteins, cH4/3 and cH4/1, consisting of an H4 head and H3 or H1 stalks respectively. These ELISAs were performed on Ni-NTA plates onto which the chimeric proteins were added. (J) Binding of mAbs to a Cal09 H1 recombinant protein. The positive control used was mAb CR9114, which is a broadly reactive human antibody binding all HA subtypes. A mAb binding to the Lassa virus glycoprotein was used as negative control. (K) IVPM. An influenza virus protein microarray was utilized to further test the breadth of the mAbs to HAs from all influenza A virus HA subtypes. Microarray slides were printed with all the respective proteins and three different dilutions per antibody were tested. Area under the curve (AUC) values from each antibody were calculated and the data is presented as a heat map. Shown are group 1 HA proteins followed by group 2 HA proteins including various H4 proteins, and a neuraminidase (N2) protein as a control. dCZ 56 corresponds to A/duck/Czechoslovakia/1956 H4, dZJ13 corresponds to A/duck/Zhejiang/D9/2013 H4, sHB 09 corresponds to A/swine/HuBei/06/2009 H4, bwtIL 10 corresponds to A/blue-winged teal/Illinois/10OS1563/2010 H4, sMO 15 corresponds to A/swine/Missouri/A01727926/2015 H4, and sDE 88 corresponds to A/shorebird/Delaware Bay/718/1988 H4. One influenza virus neuraminidase, N2, was added as an irrelevant protein and the negative control used was an anti-Lassa glycoprotein antibody.

Eight IgG antibodies were obtained via classical hybridoma fusion from a mouse immunized with cH4/3N1 and cH4/1N1 viruses which both carry the head domain of the A/duck/Czechoslovakia/1956 (H4N6) strain [27]. The isotypes of the eight mAbs are listed in Table 1. The original purpose of this work was to generate mAbs for quantification and identity testing of chimeric HA-based vaccine candidates [28] but given the importance of the H4 subtype, we decided to further characterize the isolated antibodies *in vitro* and *in vivo*. The initial screening for selection of the mAbs was performed with recombinant H4 HA from A/duck/Czechoslovakia/1956 (H4N6). As a next step, we wanted to assess the cross-reactivity of these antibodies. To analyse the differences in the hemagglutinin of various available H4 isolates and to pick representative strains for analysis, full-length amino acid sequences of these H4s were aligned using Clustal Omega and analysed via a neighbor-joining tree and visualized (Figure 1(A)). As seen, H4 HAs cluster into two different lineages, the Eurasian lineage or the North American lineage. A/duck/Czechoslovakia/1956 (H4N6), the first H4 virus isolated in 1956, classifies into the Eurasian lineage. To assess binding of the eight antibodies to the various representative recombinant H4 proteins from avian and mammalian isolates that were purified in the laboratory, an enzyme-linked immunosorbent assay (ELISA) was performed. Three H4 proteins from the Eurasian lineage were selected (Figure 1(B–D)) while another three from the North American lineage (Figure 1(E–G)) were selected for analysis via ELISA. Across the lineages, KL-H4-2B1 had a consistently high minimum binding concentration (MBC). Several antibodies like KL-H4-1G4, KL-H4-3D8, KL-H4-3G7, KL-H4-4A11, and KL-H4-5B8 showed high cross-reactivity to avian and swine isolates of both lineages. To further investigate where the antibodies are binding (antibodies were raised against the head domain of H4 using cH4/3 and cH4/1 proteins), ELISAs were performed with a chimeric protein consisting of the head domain of H4 but the stalk of either H1 (Figure 1(H)), H3 (Figure 1(I)) and a plain wild-type A/California/04/09 (Cal09) H1 (Figure 1(J)). Interestingly, while all mAbs bound both cH4/1 and cH4/3 proteins, binding of most antibodies was stronger to cH4/3 protein. The only mAb that showed cross-reactivity to H1 HA was KL-H4-5B8 while all other mAbs showed binding to H4 only, providing evidence that they bind to the head domain. Human mAb CR9114 [29], which binds to all influenza A HA subtypes (as well as influenza B HA), was used as a positive control and an irrelevant IgG (anti-Lassa virus GPC, KL-AV-1A2 [30]) was used as a negative control in the ELISA. To further investigate the cross-reactivity of the mAbs, an influenza virus protein microarray (IVPM) [31] was utilized to test binding to all HA subtypes from H1 to H18 (Figure 1(K)). In this assay KL-H4-5B8, showed reactivity to several HAs including H4, H8, H14 and H15 (but not H1 from H1N1 strain A/PR/8/34 (PR8) which was printed on the IVPM). KL-H4-3G7 also showed detectable binding to H10. As negative control a recombinant N2 NA was printed on the microarray to ensure that the detected binding was specific. No signal was detected for the negative control.

**Table 1.** Isotype and hemagglutinin inhibition activity of the eight mAbs.

Antibody	Isotype	HAI activity A/duck/Czechoslovakia/1956 H4N6-PR8	HAI activity cH4/3N1
KL-H4-1E8	IgG2b	Negative	Negative
KL-H4-1G4	IgG2a	Negative	Negative
KL-H4-2B1	IgG2a	Negative	Negative
KL-H4-3D8	IgG2b	Negative	Negative
KL-H4-3G7	IgG2b	Negative	Negative
KL-H4-4A11	IgG2a	Negative	Negative
KL-H4-4E8	IgG2b	Negative	Negative
KL-H4-5B8	IgG1	Negative	Negative

**Figure 2.**
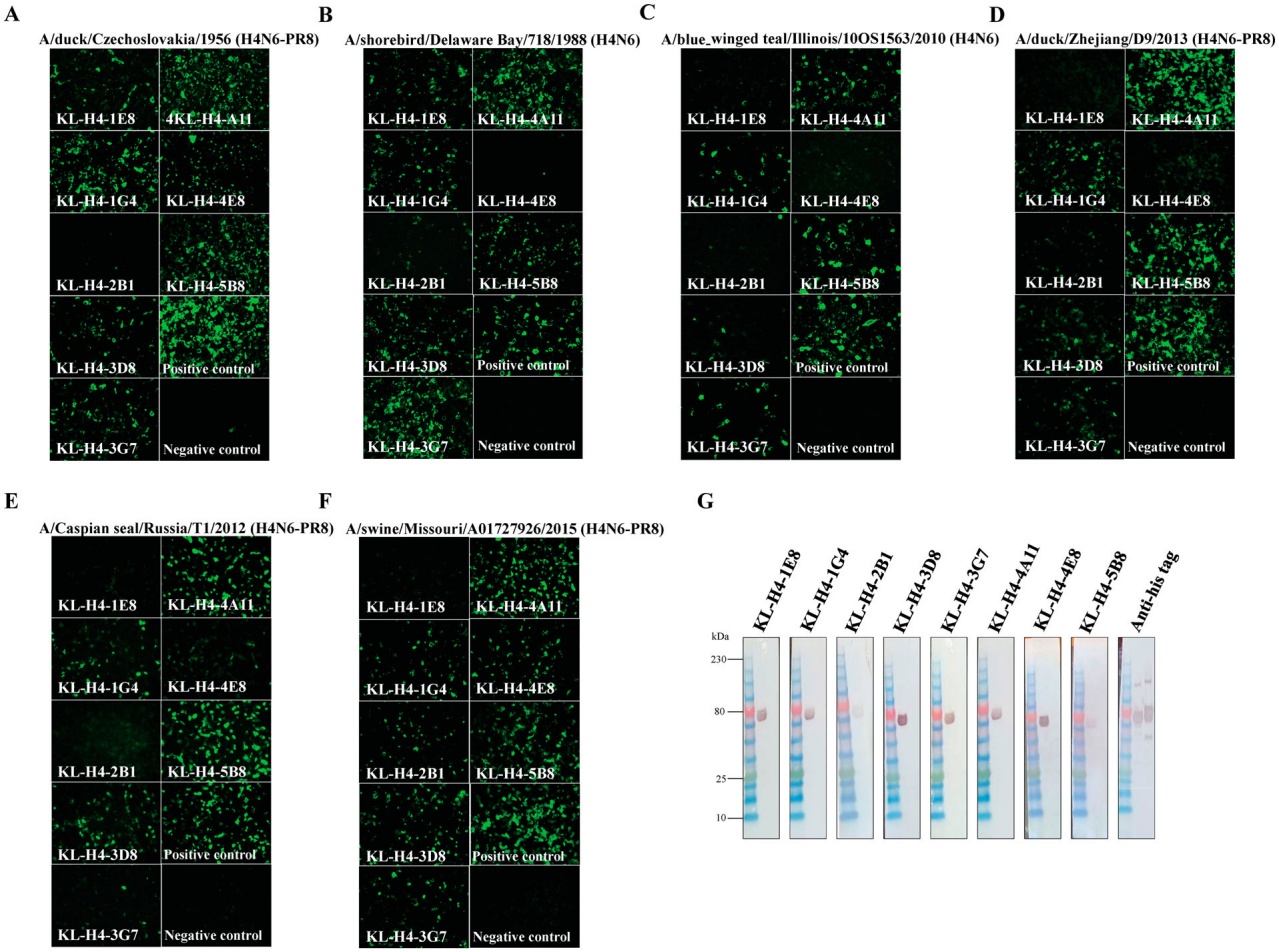
Antibody binding to H4 on infected cells and in Western blot. (A–F) Immunofluorescence analysis to assess binding of antibodies to HA on the surface of infected cells. MDCK cells were infected with (A) A/duck/Czechoslovakia/1956 (H4N6-PR8), (B) A/shorebird/Delaware Bay/718/1988 (H4N6), (C) A/blue-winged teal/Illinois/10OS1563/2010 H4N6, (D) A/duck/Zhejiang/D9/2013 (H4N6-PR8), (E) A/Caspian seal/Russia/T1/2012 (H4N6-PR8), and (F) A/swine/Missouri/A01727926/2015 (H4N6-PR8) followed by staining with 30 μg/ml of each antibody and secondary antibody treatment (anti-mouse Alexa Fluor 488). A mAb binding to the Lassa virus glycoprotein served as negative control while a pan HA mAb, CR9114, served as positive control. (G) Western blot analysis. Recombinant H4 (A/duck/Czechoslovakia/1956) and recombinant H11 HA were denatured and reduced, run on an SDS-PAGE and then blotted onto a nitrocellulose membrane. Membranes were probed with 30 μg/ml of each antibody and then treated with anti-mouse IgG alkaline phosphatase secondary antibody. Both recombinant proteins feature a hexahistidine tag, and an anti-hexahistidine antibody was used as a positive control.

Although binding via ELISA to the recombinant H4 was confirmed, we also wanted to assess whether the antibodies can bind to the H4 as it is expressed on the surface of on infected cells in its native confirmation. Madin Darby canine kidney (MDCK) cells were infected with A/duck/Czechoslovakia/1956 H4N6-PR8 (here and below “-PR8” indicates a 6:2 reassortant with HA and NA from the H4N6 strain and the internal proteins from PR8) (Figure 2(A)), A/red knot/Delaware/541/1988 (H4N6) (Figure 2(B)), A/blue-winged teal/Illinois/10OS1563/2010 (H4N6) (Figure 2(C)), A/duck/Zhejiang/D9/2013 (H4N6-PR8) (Figure 2(D)), A/Caspian seal/Russia/T1/2012 H4N6-PR8 (Figure 2(E)), and A/swine/Missouri/A01727926/2015 (H4N6-PR8) (Figure 2(F)). KL-H4-1E8 and KL-H4-2B1 had very high MBC values (low binding) across the six ELISAs and this was consistent with the immunofluorescence (IF) assay as these antibodies only bound strongly to two avian isolates but showed little to no binding to the other four viruses. Several mAbs such as KL-H4-1G4, KL-H4-4A11, and KL-H4-5B8 showed very good binding across all six IF panels. Of note, there was also a difference for some of the mAbs in terms of binding to recombinant protein in ELISA and the same HA on the infected cells hinting at small differences in the HA conformation in the two different assays. In order to investigate whether these antibodies target epitopes that are conformational or linear, Western blots were performed using recombinant H4 from A/duck/Czechoslovakia/1956 H4N6 with recombinant H11 HA as an irrelevant protein control in each blot (Figure 2(G)). Six antibodies strongly bound H4 HA in Western blot despite the protein being transferred to the membrane from a denaturing, reducing gel hinting at linear or microconformational target epitopes. MAb KL-AV-2B1, which had weak binding in ELISA and IF assays, also showed a very faint band on the Western blot. MAb KL-H4-5B8 also displayed weak reactivity perhaps indicating that its epitope is of conformational nature. Both H4 and H11 HA featured a hexahistidine tag at the C-terminus and to ensure efficient transfer on the Western blot, an anti-hexahistidine antibody was used as a positive control.

### The majority of the isolated mAbs have no neutralizing activity but are active in an ADCC reporter assay

**Figure 3.**
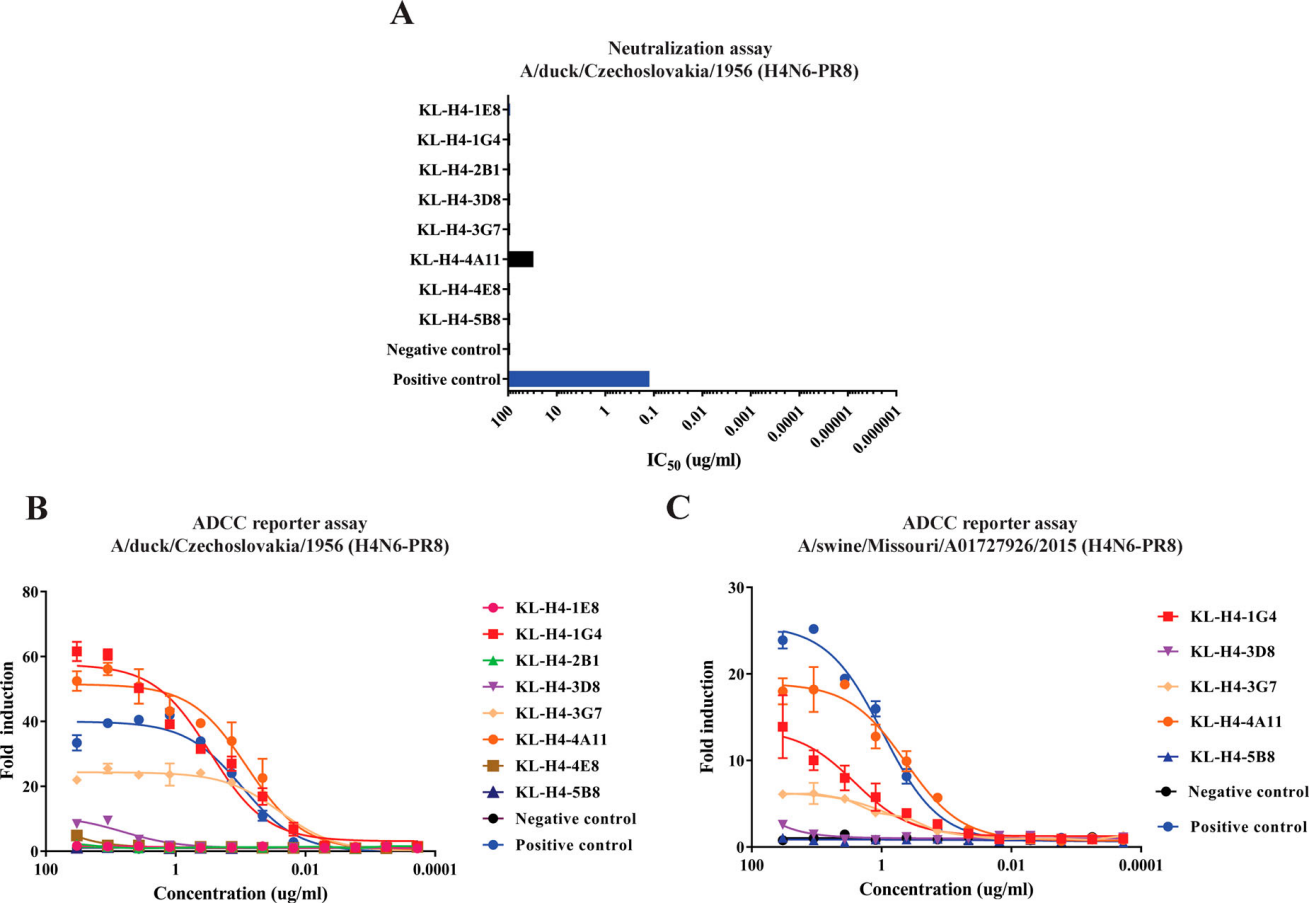
Only one mAb neutralizes *in vitro* but several antibodies show ADCC activity *in vitro*. (A) Microneutralization assay against A/duck/Czechoslovakia/1956 (H4N6-PR8). A microneutralization assay was performed using all the eight antibodies at a starting concentration of 100 μg/ml. (B–C) ADCC reporter assay to assess engagement of the murine FcγIV receptor by the antibodies bound to infected cells. MDCK cells were infected with A/duck/Czechoslovakia/1956 (H4N6-PR8) (B) or A/swine/Missouri/A01727926/2015 (H4N6-PR8), then incubated with various dilutions of each antibody and effector/reporter cells expressing luciferase upon activation. Luminescence was measured as a readout. The negative control antibody was an anti-Lassa virus glycoproteins antibody and the positive control antibody used was mAb CR9114.

To assess the *in vitro* functionality of the mAbs, a microneutralization (MN) assay was performed to test whether the antibodies are capable of inhibiting virus replication. Although all antibodies bound to the H4 of A/duck/Czechoslovakia/1956 (H4N6), only KL-H4-4A11 neutralized the virus and had a 50% inhibitory concentration (IC_50_) of 33 μg/mL (Figure 3(A)) indicating low neutralizing potency. All other antibodies failed to show any neutralization activity while the positive control, CR9114, was highly neutralizing and had an IC_50_ of approximately 1 μg/mL. In addition, a hemagglutination inhibition assay (HAI) was performed with A/duck/Czechoslovakia/1956 H4N6-PR8 but none of the antibodies showed any inhibition activity (Table 1). Several studies have recently shown that non-neutralizing antibodies can protect mice from infection due to engagement of Fc receptors and triggering of effector functions [32–34]. Hence, an *in vitro* antibody-dependent cell-mediated cytotoxicity (ADCC) reporter bioassay kit was used to assess whether these eight antibodies can engage the mouse FcγRIV receptor on effector cells as a surrogate for *in vivo* Fc-FcR based effector functions [35]. Two different viruses, A/duck/Czechoslovakia/1956 (H4N6-PR8) (Figure 3(B)) and A/swine/Missouri/A01727926/2015 (H4N6-PR8) (Figure 3(C)) were chosen to run the ADCC reporter assay. These two viruses were selected for this assay and for downstream *in vivo* characterization because one is of avian origin while the other is of swine origin and they belong to different geographic lineages. Furthermore, only antibodies that showed significant binding in ELISA to A/swine/Missouri/A01727926/2015 H4 and had low MBC values were used in the ADCC reporter assay against that virus. As shown in Figure 3(B and C), several antibodies had high ADCC reporter activity (approximately 10-fold induction of above), especially KL-H4-1G4 and KL-H4-3G7, both of which are non-neutralizing. The weakly neutralizing antibody, KL-H4-4A11, also displayed strong ADCC reporter activity. Antibodies that had ADCC reporter activity against A/duck/Czechoslovakia/1956 (H4N6-PR8), an avian virus from the Eurasian lineage, also had ADCC reporter activity (although weaker) against the other virus, A/swine/Missouri/A01727926/2015 (H4N6-PR8), which is a swine virus from the North American lineage.

### Establishment of an H4N6 mouse model that allows for lethal viral challenge

**Figure 4.**
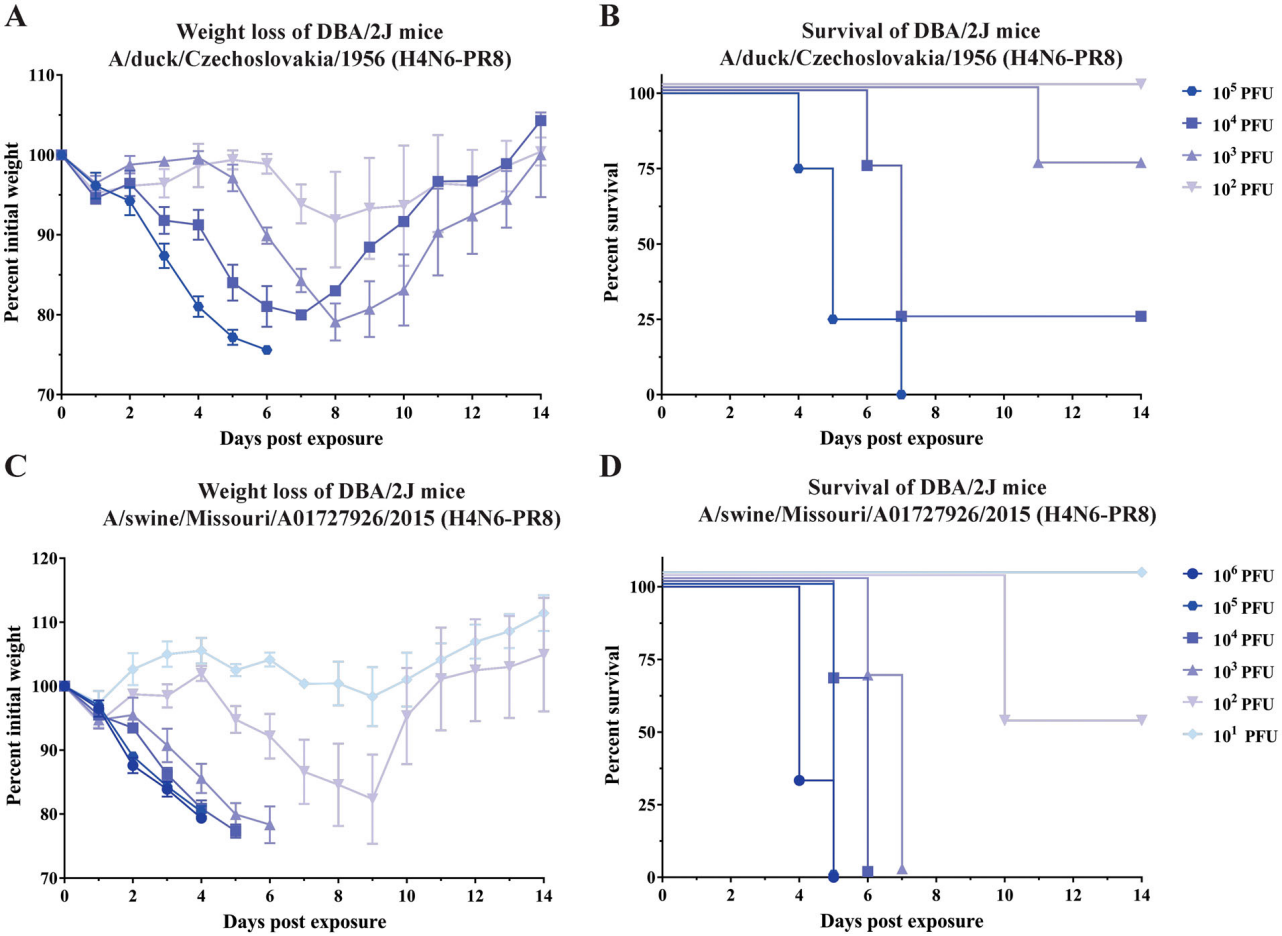
Infection of DBA/2J mice with recombinant H4N6 viruses in the PR8 background. (A) Weight loss of mice (*n* = 4) after infection with A/duck/Czechoslovakia/1956 (H4N6-PR8). DBA/2J mice were infected with various doses of A/duck/Czechoslovakia/1956 (H4N6-PR8) and monitored for 14 days after infection. (B) Survival of mice after infection with A/duck/Czechoslovakia/1956 (H4N6-PR8). DBA/2J mice were infected with various doses of A/duck/Czechoslovakia/1956 (H4N6-PR8) and weight loss was monitored for 14 days after infection. (C) Weight loss of mice after infection with A/swine/Missouri/A01727926/2015 (H4N6-PR8). DBA/2J mice were infected with various doses of A/swine/Missouri/A01727926/2015 (H4N6-PR8) and monitored for 14 days after infection. (D) Survival of mice after infection with A/swine/Missouri/A01727926/2015 (H4N6-PR8). DBA/2J mice were infected with various doses of A/swine/Missouri/A01727926/2015 (H4N6-PR8) and weight loss was monitored for 14 days after infection.

Currently, no small animal model exists to study protection from influenza viruses belonging to the H4 subtype. Wild type H4 viruses, while being able to replicate, are usually not inducing significant morbidity and mortality in mice. The PR8 backbone is adapted to mice and usually confers murine pathogenicity if used as backbone (while being safe in ferrets, humans and chickens) [36–38]. In order to investigate if the various recombinant H4N6 viruses that were rescued in the laboratory in a PR8 background (6:2 re-assortants) were lethal in mice and suitable to establish a challenge model, female BALB/c were initially used. BALB/c mice were infected with various doses of A/duck/Zhejiang/D9/2013 (H4N6-PR8) (Figure S1(A–B)) and monitored for weight loss over fourteen consecutive days post infection. As seen in the figure, mice lost body weight when infected with 10^5^ plaque forming units (PFU) of virus but gradually regained weight after day 5 post infection. One mouse in the 10^5^ PFU group succumbed to infection but 75% of the group survived. The same experiment was also repeated with A/swine/Missouri/A01727926/2015 (H4N6-PR8) in BALB/c mice. As shown in Figure S1(C), mice in the 10^6^ PFU group as well as 10^5^ PFU group lost significant amounts of body weight (Figure S1(C)). All mice succumbed to infection between day 4 and day 5 in the 10^6^ PFU group but mice recovered quickly in the other groups that received lower doses of the virus (Figure S1(D)). Thus, recombinant H4N6 viruses, even in the PR8 background, did not seem very pathogenic and did not lead to significant lethality in BALB/c mice. Hence, similar experiments were then performed using DBA/2J mice which have been reported to be more susceptible to influenza virus infections [39,40]. In these experiments, we decided to use the same viruses that were used for the ADCC reporter assay because A/duck/Czechoslovakia/1956 (H4N6-PR8) is an avian Eurasian lineage virus while the A/swine/Missouri/A01727926/2015 (H4N6-PR8) is of swine origin and belongs to the North American lineage. DBA/2J mice (*n* = 4 per group) were infected with various doses of A/duck/Czechoslovakia/1956 (H4N6-PR8) and weight loss (Figure 4(A)) and survival (Figure 4(B)) was monitored for fourteen days post infection. Mice lost significant amounts of body weight in groups that received 10^5^, 10^4^, and 10^3^ PFU of the virus. All mice succumbed to infection in the 10^5^ PFU group while the group that received 10^4^ PFU of virus had 3 mice dead out of 4 (25% survival), as seen in Figure 4(B). The group that was infected with 10^3^ PFU of virus had just one casualty with 75% survival. The murine 50% lethal dose (mLD_50_) was calculated and found to be 2.9 × 10^4^ PFU. Infection with the swine H4 virus, A/swine/Missouri/A01727926/2015 (H4N6-PR8), was more severe as all groups had significant weight loss except the group that was infected with 10^1^ PFU (Figure 4(C)). In terms of survival, groups infected with 10^6^, 10^5^, 10^4^ and 10^3^ PFU of the virus showed 0% survival. Mice in the 10^2^ PFU group had 50% survival while all mice survived the infection in the 10^1^ PFU group (Figure 4(D)). The mLD_50_ of this virus was found to be 5 × 10^2^ PFU. Based on the mLD_50_, the swine virus is more lethal than the avian virus. One potential explanation for this is, that the HA of the swine virus is better able to interact with host receptors found in mice.

### Broadly reactive anti-H4 mAbs protect mice from lethal H4N6 challenge with strains from the Eurasian and North American lineage despite the absence of strong neutralizing activity

**Figure 5.**
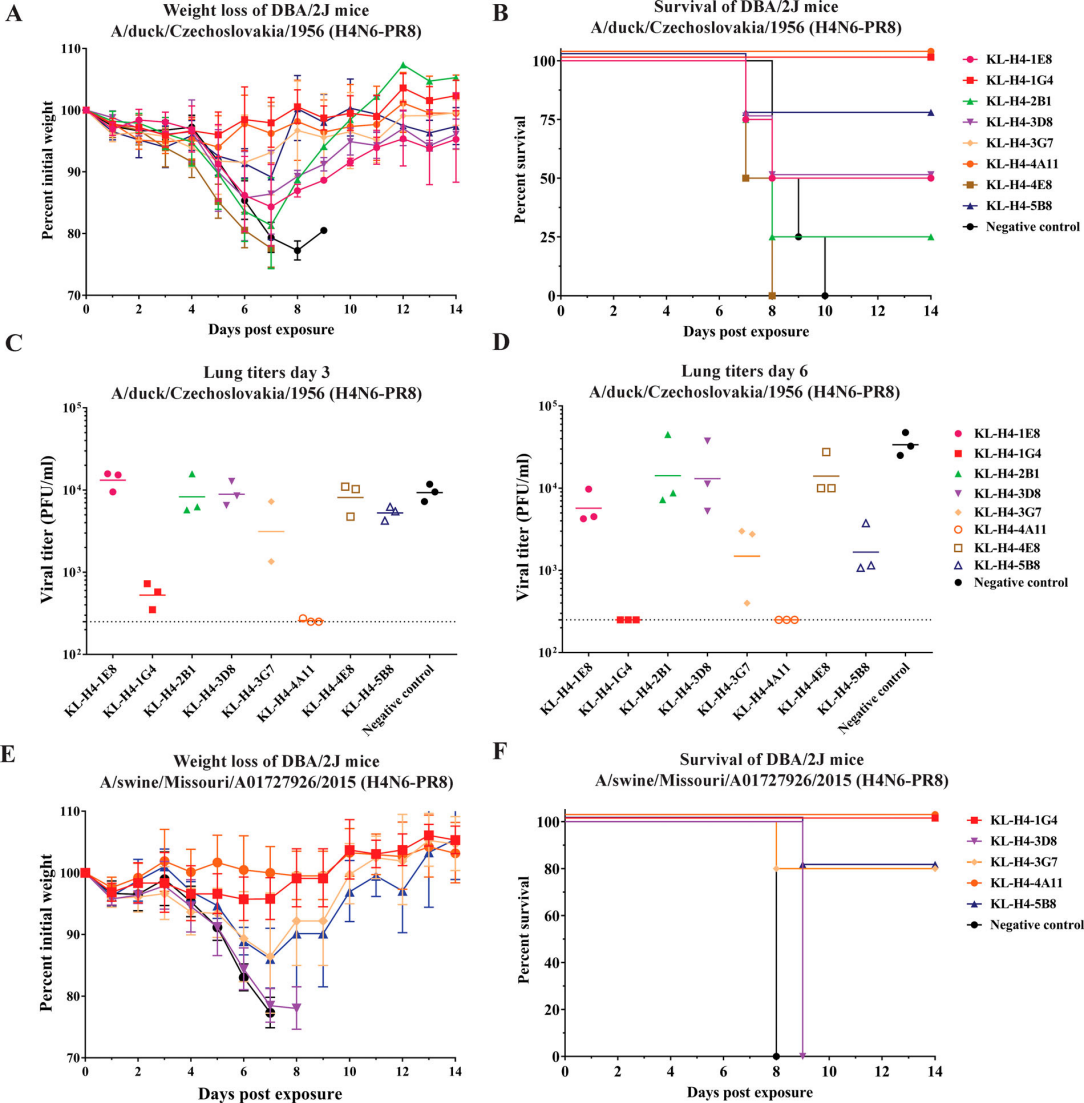
Protective prophylactic efficacy of mAbs in DBA/2J mice against challenge with A/duck/Czechoslovakia/1956 (H4N6-PR8) or A/swine/Missouri/A01727926/2015 (H4N6-PR8). (A–B) Mice were administered 10 mg/kg of each mAb intraperitoneally two hours prior to intranasal challenge with 5 mLD_50_ of A/duck/Czechoslovakia/1956 (H4N6-PR8) virus and mice were monitored for 14 days after infection. Survival (A) and weight loss (B) curves are shown. The negative control antibody was an anti-Lassa virus glycoprotein antibody. (C–D) Viral lung titres after infection of DBA/2J mice with A/duck/Czechoslovakia/1956 (H4N6-PR8). Ten mg/kg of each respective antibody was administered intraperitoneally two hours prior to intranasal infection with 1 mLD_50_ of virus. Mice were sacrificed on day 3 (C) and day 6 (D) and lungs were harvested and viral titre was determined using a plaque assay. (E-F). Mice were administered 10 mg/kg of mAb intraperitoneally two hours prior to intranasal challenge with 5 mLD_50_ of A/swine/Missouri/A01727926/2015 (H4N6-PR8) virus and mice were monitored for 14 days after infection. Survival (E) and weight loss (F) curves are shown. The negative control antibody was an anti-Lassa virus glycoprotein antibody.

Having established a mouse model for H4 viruses, the next experiments revolved around testing whether the eight mAbs have any *in vivo* protective effect in DBA/2J mice in a prophylactic setting. Using the mLD_50_ calculated in the previous figure, 10 mg/kg of antibody was administered intraperitoneally and mice were infected with 5 mLD_50_ of A/duck/Czechoslovakia/1956 (H4N6-PR8) via the intranasal route 2 h later. Weight loss (Figure 5(A)) and survival (Figure 5(B)) of mice in each antibody group were assessed. Two mAbs, KL-H4-2B1 and KL-H4-4E8, behaved similar to the negative control group and antibody administration seemed to have little to no effect. However, mice treated with mAbs KL-H4-1G4, KL-H4-3G7 or KL-H4-4A11 showed little to no body weight loss indicating good protection from challenge. Survival was also assessed and as seen in Figure 5(B), administration of KL-H4-1G4, KL-H4-3G7, and KL-H4-4A11 led to 100% survival of the mice. Meanwhile, all animals in the group that received KL-H4-4E8 succumbed to infection by day 8 and mice in the KL-H4-2B1 group showed only 25% survival.

In addition to assessing weight loss and survival, we also wanted to investigate if administration of mAbs could reduce the viral load in the lungs of mice in a prophylactic setting. The same experiment as above was performed except that mice were euthanized and plaque assays using lung homogenates were performed to assess viral titre on day 3 (Figure 5(C)) and day 6 (Figure 5(D)) post infection. A/duck/Czechoslovakia/1956 (H4N6-PR8) was used as challenge virus and the challenge dose was lowered to 1 mLD_50_ to increase sensitivity of the assay. The results of this experiment mirrored what was observed in the earlier experiment with the same virus. Groups receiving KL-H4-1G4 and KL-H4-4A11 had very low virus titres in the lungs on day 3 and no virus was detected for these two groups on day 6. It is interesting to see that KL-H4-5B8 was also able to reduce viral titres on day 6 post infection when compared to the negative control group since it led to 75% survival in the earlier experiment. Unfortunately, one mouse from KL-H4-3G7 died after anaesthesia treatment on day 0 of the experiment (day 3 group) but this antibody group also showed reduced viral titre in the lungs on day 6. Next, we also tested the efficacy of our antibodies with a swine H4 virus from a distinct lineage, A/swine/Missouri/A01727926/2015 (H4N6-PR8), in a prophylactic setting using a 5 mLD_50_ challenge dose. As described above, weight loss (Figure 5(E)) and survival (Figure 5(F)) of mice from each group was monitored for fourteen days post infection. Here again, only antibodies that showed strong reactivity in ELISA were used. As observed with the avian H4N6 virus, KL-H4-1G4 and KL-H4-4A11 treatment led to minimal weight loss which translated to 100% survival. Meanwhile, mice that received KL-H4-3G7 and KL-H4-5B8 lost up to 15% initial body weight but gradually recovered after day 7. Only one mouse succumbed to infection from each of these two groups resulting in 80% overall survival. These data show that epitopes conserved among avian and swine viruses of the H4 subtype exist which can be targeted by antibodies that confer protection *in vivo*.

Since KL-H4-4A11 was the only neutralizing mAb, the ADCC reporter assay was also performed using combinations of all other mAbs with KL-H4-4A11 to see if an additive or synergistic effect can be observed (Figure S2(A)). However, no marked increase above the activity of KL-H4-4A11 was detected. In addition, we also tested combinations of one mAb, KL-H4-1G4, with KL-H4-4A11 to see if increase protection is observed in vivo. However, although the mAb dose was lowered to 3 mg/kg, no differences in weight loss between the different groups was observed and 100% survival was recorded for all groups (Figure S2(B and C)).

## Discussion

Viruses of the H4 subtype are widespread in avian species, infect mammals and serological evidence for exposure to these viruses can be found in humans who work closely with poultry [10,12–17,22,23]. In addition, H4 HA can bind quite well to α-2,6-linked sialic acid, specifically HA of mammalian isolates, and also tolerates the insertion of a polybasic cleavage site into the HA which leads to an HPAI phenotype in experimentally infected birds [13,20,21,24]. Therefore, while not of immediate pandemic concern, it is worth learning more about the antigenicity of H4 HA. The first H4N6 virus (then called Hav4 Nav1) was isolated in 1956 and follow up work in the 1980s with monoclonal antibodies led to the identification of 3 antigenic sites, A, B and C [41,42]. Interestingly, during this early work with HAI active mAbs, it was already shown that H4 HAs can be antigenically quite conserved. However, little work has been performed since then to further investigate the antigenicity of H4 and the protective potential of antibodies against it. The mAbs characterized here are drastically different than the ones isolated in the 1980s since only one has neutralizing activity at a very high concentration (KL-H4-4A11) and none of the mAbs have HAI activity.

Importantly, many of the mAbs showed cross-reactivity to H4 HAs from viruses of both the Eurasian lineage and the American lineage including avian and mammalian isolates, suggesting that they bind to relatively conserved epitopes. Interestingly, binding was best towards the cH4/3 HA, which was the first immunogen that was used to generate the mAbs. Lower binding for some of the mAbs to wild type H4 HAs and even a cH4/1 HA (which features exactly the same head domain as the cH4/3 protein) was observed. The reason for this could be that the structure of the HA head domain on cH4/3 is slightly different than the head domain on wild type H4 HA or cH4/1 HA. This was observed in the past for a cH5/1N1 construct and could potentially explain this discrepancy [43]. Another interesting finding was that one mAb, KL-H4-5B8, showed cross-reactivity to H8, H14 and H15 in IVPM suggesting heterosubtypic binding. Interestingly, this mAb also bound to Cal09 H1 HA in ELISA but not to PR8 H1 HA on the IVPM. The explanation for this could be that the mAb targets one of the described cross-reactive head trimer-interface epitopes [44] that are only accessible in loose trimer conformation, as is known for Cal09 H1 HA [45–47]. An alternative explanation could be that the sequence of this epitope is present on Cal09 H1 and H4, H8, H14 and H15 HAs but not PR8 H1 HA.

To test the potentially protective effect of these mAbs *in vivo*, we developed a mouse model based on HA and NA from an avian and a swine H4N6 isolate in a PR8 backbone. These viruses were lethal in the mouse model. Treatment with 5 out of 8 mAbs led to considerable protection (50–100% survival) in this model, despite the fact that 4 of the 5 protective mAbs did not show any neutralizing activity. This can likely be attributed to the mAbs’ Fc-effector functions. In fact, the mAbs that protected to the greatest extent showed high activity in an ADCC reporter assay. Of note, KL-H4-5B8, which showed partial protection (75% survival), had no activity in this assay and was of the IgG1 isotype which is not expected to interact strongly with activating FcRs in mice. Therefore, other effector functions might have led to protection by this mAb. Our data suggest that H4 HAs are antigenically relatively conserved and that potential vaccine candidates might induce antibodies that can protect from a broad range of H4 viruses. Of note, these data are derived from mAbs that were isolated from a single mouse and therefore additional studies are needed before firm general conclusions about cross-protection of H4 antigens can be drawn. The animal models established here will facilitate future H4 virus research.

## Materials and methods

### Cells and viruses

Madin-Darby canine kidney (MDCK) cells (ATCC #CCL-34) were passaged in Dulbecco’s Modified Eagles Medium (complete DMEM, Gibco) which was supplemented with 10% fetal bovine serum (FBS, HyClone) and antibiotics solution consisting of 10,000 units per mL of penicillin and 10,000 µg/mL of streptomycin (Pen Strep, Gibco). HA and NA sequences from the following isolates were obtained from GenBank and were commercially synthesized (Invitrogen GeneArt Gene Synthesis): A/duck/Czechoslovakia/1956 (H4N6, M25283 and GU052383), A/duck/Zhejiang/D9/2013 (H4N6; KT589226 and KT589272), A/swine/HuBei/06/2009 (H4N1; JX878675 and JX878677), A/Caspian seal/Russia/T1/2012 (H4N6; KJ847700 and KJ847705) and A/swine/Missouri/A01727926/2015 (H4N6; KT589226 and KT589272). Recombinant H4N6 viruses were rescued with the HA and NA from the original isolate and the remaining six segments from A/PR/8/34 (PR8) as 6:2 re-assortant viruses using the protocol described previously [48]. These recombinant viruses are labelled with PR8 in the name. For instance, A/duck/Czechoslovakia/1956 (H4N6) is labelled as A/duck/Czechoslovakia/1956 (H4N6-PR8) in all the figures. In addition, chimeric HA expressing viruses were used. These viruses express globular head domains from H4 HA and stalk domains from H1 or H3 HAs. The head region is defined as the region between C52 and C277 (H3 numbering) and was derived from A/duck/Czechoslovakia/1956 (H4N6). The remaining part of the HA including the signal peptide, the stalk domain, the transmembrane domain and the cytoplasmic tail were derived from either H1 (A/California/04/09) or H3 (A/Perth/16/09) HA. These viruses included cH4/1N1 (6:2 re-assortant, virus in a PR8 background, N1 from A/California/04/09 [27]) and cH4/3N1 virus (7:1 re-assortant virus in the PR8 background [27]). A/blue-winged teal/Illinois/10OS1563/2010 (H4N6; BEI Resources # NR-35982) and A/red knot/Delaware/541/1988 (H4N6; BEI Resources # NR-45153) were obtained from the Biodefense and Emerging Infections Research Resources Repository (BEI Resources). Viruses were grown in 10-day-old embryonated chicken eggs (Charles River Laboratories) and viral titres were assessed on MDCK cells via a standard plaque assay as described [49].

### Recombinant HA and NA glycoproteins

All recombinant proteins utilized were expressed and purified via the baculovirus expression system as described in detail in the literature [50,51]. The ectodomains of the HA proteins from the following viruses were cloned into a baculovirus shuttle vector: A/duck/Czechoslovakia/1956 (H4N6), A/duck/Zhejiang/D9/2013 (H4N6), A/swine/HuBei/06/2009 (H4N1), A/swine/Missouri/A01727926/2015 (H4N6), A/blue-winged teal/Illinois/10OS1563/2010 (H4N6), and A/red knot/Delaware/541/1988 (H4N6) as well as cH4/1 and cH4/3 HAs. This shuttle vector contains a C-terminal trimerization domain as well as a hexahistidine tag [51]. Baculoviruses were propagated in Sf9 cells and these viruses were then used to infect BTI-TN-5B1-4 cells. Proteins were purified from the supernatant using a standard protocol [50,51] and used appropriately as required per assay.

### ELISA

Ninety-six-well plates (Immulon 4 HBX; Thermo Scientific) were coated overnight at 4°C with 50 μL of purified protein per well at a concentration of 2 μg/mL diluted in coating solution (10X Coating Solution Concentrate diluted 1:10; Seracare). The next morning, the coating solution was removed and 100 μL per well of 3% non-fat milk prepared in phosphate buffered saline with 0.1% Tween 20 (TPBS) were added for an hour at room temperature (RT). Next, primary antibody solutions were prepared in TPBS containing 1% non-fat milk starting at 10 μg/ml followed by 1:3 dilutions. 100 μL of each dilution was added onto the coated plates for an hour at RT. Plates were washed three times with 100 μL per well of TPBS. Next, 100 μL per well of a horseradish peroxidase (HRP)-labeled anti-mouse IgG (GE Healthcare) was added to the plates. This secondary antibody was diluted 1:3,000 in 1% non-fat milk in TPBS. For human antibodies (e.g. CR9114), goat anti-human IgG-HRP (ThermoFisher Scientific) was used at the same dilution. After one hour of incubation at RT, plates were washed thoroughly three times with TPBS. Hundred micro litre per well of SigmaFast OPD (*o*-phenylenediamine dihydrochloride; Sigma-Aldrich) solution was added to the plates. This substrate was left on the plates for 10 min after which 50 μL per well of 3 M hydrochloric acid (HCl) was added onto the plates to halt the reaction. Using a Synergy 4 (BioTek) plate reader, the reactivity was measured at an optical density of 490 nm. The minimal binding concentration was calculated as the last dilution of antibody which gave a signal that was above the background blank value (of wells treated with secondary antibody only). The blank value was calculated by averaging the blank wells on the plate plus three standard deviations. Data were analysed using the software Prism 7 (Graphpad). The ELISAs in Figure 1(H and I) were performed using Ni-NTA HisSorb Plates (Qiagen). Fifty micro litre of a 2 μg/mL solution of recombinant protein was added onto the plates suspended in PBS. After an hour, this was removed and the ELISA assay was continued using the same exact protocol discussed above.

### Influenza virus protein microarray (IVPM)

Recombinant HA was spotted onto Nexterion E epoxysilane-coated glass slides (Schott, Mainz, Germany) in arrays of 25 spots. The arrays were comprised of eight HAs, printed in triplicate and spotted in volumes of 30 nL per spot and at a concentration of 100 μg/mL in PBS. Each glass slide included 24 separate arrays. Each slide was incubated for 90 minutes at >95% relative humidity in a sealed chamber at room temperature after printing, and stored for 14–18 h in a vacuum-sealed package before blocking. Slides were blocked with 3% non-fat milk in PBST for one hour, then washed in PBST by immersion before being inserted into 96-well microarray gaskets (Arrayit, Sunnyvale, CA, USA). Antibodies were diluted to starting dilutions of 30 μg/mL in 1% non-fat milk PBST, and were then added to the array and serially diluted two times at 1:10 into separate arrays. Antibodies were incubated with the arrays for one hour, then the antibodies were removed and the arrays were washed three times with 220 μL PBST/array. Then, each 50 μL secondary antibody solution was added to and incubated with each array for one hour. The secondary antibody solution was then removed, and the arrays were washed three times with 220 μL/array PBST, rinsed in deionized water, and with an air compressor. Slides were then analysed for mean fluorescence with a Vidia microarray scanner (Indevr, Boulder, CO, USA), at an exposure time of 1000 ms. Area under the curve (AUC) was measured as total peak above 0.04. The technique has been described in detail in [31].

### Generation and selection of hybridomas secreting H4 antibodies

One 6–8 weeks old, female, BALB/c mouse (Jackson Laboratories) was injected via the intraperitoneal route with 10^6^ plaque forming units (PFU) in 100 μL of PBS of a cH4/3N1 virus (7:1 re-assortant virus in the PR8 background [27]). Three weeks later, the mouse was intransally infected with the same virus at the same dose but in a smaller volume (50 μL). Four weeks later, the mouse was injected intraperitoneally with 100 μg of purified live cH4/1N1 (6:2 re-assortant, virus in a PR8 background, N1 from A/California/04/09 [27]) with addition of 10 μg of poly(I:C). After three days, the mouse was euthanized and the spleen was removed. Splenocytes were then fused with SP2/0 myeloma cells using polyethylene glycol (PEG, Sigma-Aldrich P7306, molecular weight 1450) and the resulting fused cells were grown on selection medium (Clonacell-HY Medium D; StemCell Technologies). After 10–14 days, single, separated colonies visible to the naked eye were picked and grown in 96-well cell culture plates. This protocol has been described in detail in [30,52,53]. Supernatant from each individual colony was screened via ELISA (described above) to see if the antibody being secreted by the hybridoma was specific to the desired antigen, H4 HA. Hybridoma lines were tested for isotype of antibody being secreted using a Pierce rapid antibody isotyping kit (Life Technologies). Selection of single, separated colonies assures that monoclonal cultures are obtained and results from isotyping confirmed the absence of any colonies producing more than one different heavy and light chain type. Selected IgG-secreting monoclonal hybridoma lines were further expanded using Medium E (StemCell Technologies) and later adapted to a serum free medium, Hybridoma SFM (Gibco). Once a hybridoma line was selected for its ability to bind the H4 protein, the respective hybridoma line was grown to a larger culture volume by repeated 1:3 splits. At the end, the supernatant was collected from each hybridoma culture and the antibody was purified. Purification was performed as per an earlier published protocol [54]. The original purpose of this work was to develop reagents for identity testing and quantification of chimeric HA expressing viruses with an H4 head domain [28].

### Immunofluorescence (IF) assay

MDCK cells were seeded on a 96-well cell culture plate at a density of 50,000 cells per well. Each respective virus was diluted in 1x minimum essential medium (MEM; Gibco). Cells were infected with a multiplicity of infection (MOI) of 1 overnight. The next day, cells were fixed with 3.7% paraformaldehyde (PFA) for an hour at room temperature. Next, cells were blocked by addition of 100 μL per well of 3% non-fat milk prepared in PBS. The blocking solution was removed and 30 μg/mL of each antibody prepared in 1% non-fat milk in PBS (100 μL per well) was added onto the cells for one hour at RT. Primary antibody was removed and the cells were washed three times with PBS. Hundred micro litre of secondary antibody, goat anti-mouse IgG heavy plus light chain (H + L)–Alexa Fluor 488 (Abcam), which was also prepared in 1% non-fat milk in PBS, was added to the cells afterwards at a dilution of 1:1000 for an hour. Cells were again washed with 100 μL per well of PBS three times. Finally, 100 μL per well PBS was added to prevent drying out of cells. Immunofluorescence was observed using a fluorescent microscope (Olympus IX-70) and images were taken and labelled.

### Western blot analysis

Ten ng of recombinant H4 HA and H11 HA (negative control) prepared in PBS were mixed with equal volume of 2x Laemmli loading buffer (Bio-Rad) supplemented with 2% beta-mercaptoethanol (BME) and heated at 100°C for 15–20 min. Samples were then run on sodium dodecyl sulphate (SDS) polyacrylamide gels (5–20% gradient; Bio-Rad) and later transferred onto nitrocellulose membranes. The remaining procedure was directly adapted from a published protocol [55]. Membranes were blocked with 3% non-fat milk in TPBS and then stained with 30 μg/mL of each antibody prepared in 1% non-fat milk in TPBS. Membranes were then washed three times with TPBS and stained with anti-mouse IgG (whole molecule)–alkaline phosphatase (AP) antibody produced in goat (Sigma-Aldrich). Reactivity was visualized by treatment of membranes with development solution prepared from AP conjugate substrate kit (Bio-Rad).

### Calculation of murine 50% lethal dose (mLD_50_)

As recommended per institutional guidelines, a mixture consisting of 0.15 mg/kg ketamine and 0.03 mg/kg xylazine in water was used as anaesthesia for mouse experiments. Various doses of each virus were prepared in sterile PBS (typically 10^6^, 10^5^, 10^4^, 10^3^, 10^2^, and 10^1^ PFU per 50 μL). Six to eight week old, female, BALB/c or DBA/2J mice (Jackson Laboratories) were split into groups of 3–4 mice. Under anaesthesia (100 μL injected intraperitoneally), each mouse was infected intranasally with the respective dose of virus (in 50 μL of PBS) and weighed on the day of infection (day 0). The weight of each mouse was then monitored daily for 14 days. Animals that had lost 25% (humane endpoint) of their initial weight were euthanized and scored dead. The murine lethal dose 50 (mLD_50_) was then calculated using the method of Reed and Muench [56].

### *In vivo* challenge studies

To assess *in vivo* efficacy of antibodies, female 6–8 weeks old DBA/2J mice (4–5 per group) were each injected with mAb at a dose of 10 mg/kg (in 100 μL of PBS) intraperitoneally. Two hours post antibody administration, mice were given anaesthesia and the day 0 weight was recorded. Next, each mouse was intranasally infected with 5 mLD_50_ of virus. Mice were then monitored daily for weight loss. As mentioned above, loss of 25% of initial body weight led to humane euthanization of the mouse and the mouse was scored dead. Weight loss and survival data were analysed in Prism 7. To assess inhibition of virus replication in the lungs, antibodies were used at a concentration of 10 mg/kg. Two hours post administration of each antibody (5–6 mice per group), mice were given anaesthesia and infected with 1 LD_50_ of virus. A lower virus dose was used to increase sensitivity of this assay. Three mice from each group were sacrificed on day 3 while the remaining three mice were euthanized on day 6. Lungs from each mouse were harvested and homogenized using a BeadBlaster 24 (Benchmark) homogenizer. Lung homogenates were frozen and were then analysed in a standard plaque assay on MDCK cells to assess viral titres.

### *In vitro* ADCC reporter assay

Using the Promega ADCC reporter bioassay kit and a published protocol, *in vitro* engagement of the ADCC receptor was assessed [35]. MDCK cells were infected at a multiplicity of infection of 1 overnight with each respective virus, as mentioned earlier in the IF assay section. The next morning, antibody dilutions were added onto the cells in addition to 75,000 effector cells per well. Cells were then left in the 37°C incubator for six hours. The luciferase substrate was added in the dark and the luminescence activity was read after 5 min using Synergy Hybrid Reader (BioTek). Anti-stalk mAb CR9114, which has known ADCC reporter activity and binding to H4 HA, was used as a positive control [29,57,58]. Fold induction over an irrelevant antibody (anti-Lassa GPC) was calculated and data were analysed in Prism 7.

### Microneutralization (MN) assay

MDCK cells were seeded in a 96-well cell culture plate (50,000 per well). Two-fold antibody dilutions starting at 100 μg/mL were prepared in 1X MEM. Virus was prepared in 1x MEM such that there were 200 PFU/50 μL. Antibody dilutions and virus were incubated together at RT on a shaking incubator for one hour. MDCK cells were washed with PBS once and the virus plus antibody mixture was added onto the cells for an hour at 37°C. The same antibody dilutions from the earlier step were prepared in 1x MEM containing tosyl phenylalanyl chloromethyl ketone-treated trypsin (TPCK- treated trypsin) at a concentration of 1 μg/mL. The virus-antibody mixture was removed from the cells. Then, antibody dilutions at the respective concentrations were added. The cells were then incubated for 48 h. After the incubation period, a hemagglutination assay was performed to test for presence or absence of virus at each well. This protocol was adapted from [59].

### Hemagglutination inhibition assay (HAI)

In order to assess whether antibodies were capable of inhibiting hemagglutination, antibody dilutions were prepared starting at 30 μg/mL and three-fold thereafter. A standard hemagglutination assay was performed to quantify the hemagglutination units (HAU) of the virus. Twenty-five μL of each antibody dilution was incubated with 8 HAU of the A/duck/Czechoslovakia/1956 (H4N6-PR8) or cH4/3N1 virus suspended in 25 μL PBS for an hour. After an hour, 50 μL of 0.5% chicken red blood cells (Lampire Biological Laboratories) per well were added on top of the virus-antibody mixture. Plates were then incubated for 45 mins to 1 h at 4°C and later analysed. This protocol has been previously published by Klausberger and colleagues [49].

### Phylogenetic analysis

Amino acids sequences of the respective HA proteins were obtained from GenBank. Alignment of the sequences was performed using Clustal Omega and the phylogenetic tree was built using the neighbor-joining tree method. The tree was finally visualized and labelled using Figtree v1.4.1.3.
